# FACTORS ASSOCIATED WITH PERCEPTION OF PATIENT-GENERATED HEALTH DATA AMONG OLDER PATIENTS POST LUNG CANCER SURGERY

**DOI:** 10.1093/geroni/igae098.3550

**Published:** 2024-12-31

**Authors:** Mona Choi, Hyeonmi Cho, Yesol Kim, Yeonju Kim

**Affiliations:** Yonsei University, Seoul, Republic of Korea; Yonsei University, Seoul, Republic of Korea; Yonsei University, Seoul, Republic of Korea; Yonsei University, Seoul, Republic of Korea

## Abstract

This study examines factors related to the perceived usefulness of patient-generated health data (PGHD) among older patients who underwent lung cancer surgery. This descriptive cross-sectional study involved individuals aged ≥65 years who had lung cancer surgery at least 4 weeks prior. Participants were recruited from an outpatient clinic, and survey data from 102 patients were analyzed. Thirty-seven PGHD items were categorized into 7 groups: Environmental Factors (EF), Lifestyle Factors (LF), Body Composition and Activity Level (BCAL), Medication-related Factors (MF), Physiological Parameters (PP), Physical and Mental Well-being Factors (PMWF), and Digital Engagement (DE). Stepwise linear regression models were used to identify factors associated with patients’ perceptions of PGHD. Higher digital literacy was associated with greater perceived usefulness in EF and DE categories. Older individuals perceived PGHD in LF and BDAL categories as less useful. Unemployed individuals found PGHD less useful in LF compared to employed individuals. Higher household income was associated with greater perceived usefulness in PMWF category. Non-smartphone users perceived PGHD as less useful in BCAL and PP categories. Patients who underwent surgery more than 5 years ago found PGHD less useful in MF and PP categories than those with more recent surgeries. Individuals with lower education levels and those living alone showed lower perceived usefulness in PP compared to their counterparts. Understanding patients’ personal characteristics, digital literacy, and technology access is crucial for utilizing PGHD effectively for health promotion among older patients with lung cancer surgery. This understanding can assist healthcare providers in identifying and addressing potential challenges.

